# CAVE-2 (Cetuximab-AVElumab) mCRC: A Phase II Randomized Clinical Study of the Combination of Avelumab Plus Cetuximab as a Rechallenge Strategy in Pre-Treated *RAS*/*BRAF* Wild-Type mCRC Patients

**DOI:** 10.3389/fonc.2022.940523

**Published:** 2022-06-27

**Authors:** Stefania Napolitano, Giulia Martini, Davide Ciardiello, Massimo Di Maio, Nicola Normanno, Antonio Avallone, Erika Martinelli, Evaristo Maiello, Teresa Troiani, Fortunato Ciardiello

**Affiliations:** ^1^ Oncologia Medica, Dipartimento di Medicina di Precisione, Università degli Studi della Campania “L. Vanvitelli”, Napoli, Italy; ^2^ Oncologia Medica, Ospedale Casa Sollievo della Sofferenza, San Giovanni Rotondo, Italy; ^3^ Dipartimento di Oncologia, Università di Torino, Azienda Ospedaliera Mauriziana, Torino, Italy; ^4^ Biologia Cellulare e Bioterapie, Istituto Nazionale per lo Studio e la Cura dei Tumori “Fondazione Giovanni Pascale”—Istituto di Ricovero e Cura a Carattere Scientifico (IRCCS), Napoli, Italy; ^5^ Oncologia Medica, Istituto Nazionale per lo Studio e la Cura dei Tumori “Fondazione Giovanni Pascale”—IRCCS, Napoli, Italy

**Keywords:** mCRC, rechallenge, cetuximab, avelumab, phase II trial

## Abstract

**Introduction:**

Immunotherapy has limited efficacy in metastatic colorectal cancer (mCRC). Understanding mechanisms mediating immune resistance in microsatellite stable (MSS) colorectal tumors remains an ongoing challenge. Novel combination immunotherapy-based approaches have been developed under the rationale of overcoming immune resistance and developing effective immune response against colorectal tumor cells. Preclinical studies have demonstrated that cetuximab may modulate immune response to cancer cells. In this scenario, the inhibition of PD-L1 with IgG1 MAb avelumab in combination with anti-EGFR IgG1 monoclonal antibody cetuximab could be a strategy for potentiating antitumor activity. The CAVE phase II single-arm clinical trial provided the first evidence of clinical activity of combining cetuximab plus avelumab in 77 patients with *RAS* wild-type (WT) mCRC. This combination had a good toxicity profile, with a low rate of common grade 3 adverse events.

**Patients and Methods:**

Based on results obtained with the CAVE clinical trial, here we describe the design and rationale for the phase II, randomized CAVE 2 clinical study of the combination of avelumab plus cetuximab as a rechallenge strategy in pre-treated *RAS*, *BRAF* WT mCRC patients treated in first line with chemotherapy in combination with cetuximab and who have had a clinical benefit (complete or partial response) from treatment. A total of 173 patients will be randomized (2:1) to cetuximab + avelumab (115) or cetuximab as a single agent (58). The primary endpoint is overall survival. Key secondary endpoints include overall response rate, progression-free survival, and safety. For each patient, before treatment, a blood sample will be obtained and analyzed for circulating free tumor DNA according to NGS (Foundation/Roche), to identify *RAS*/*BRAF* WT patients to be enrolled. The same procedure will be performed at the progression of the disease. Additional blood/plasma, tumor, and fecal samples will be collected and centrally stored for additional translational studies.

**Discussion:**

This study will provide the rationale to test immunotherapy-based combinations in the clinical setting, offering new opportunities for *RAS* WT mCRC patients.

**Clinical Trial Registration:**

[https://clinicaltrials.gov/ct2/show/NCT05291156], identifier [NCT05291156].

## Introduction

Colorectal cancer (CRC) is the third most commonly diagnosed cancer in men and the second in women, with 1.9 million new cases and 0.9 million of deaths in 2020 according to the World Health Organization (WHO). The prevalence of CRC showed a significant increasing trend in recent years, representing 10% of the global cancer incidence and 9.4% of all cancer-caused deaths (1, 2). Although the advances in screening and medical treatments have led a trend in reduction of both incidence and mortality, almost 20% of patients present metastases at the time of diagnosis, and approximately 35% of patients will subsequently develop a metastatic disease (3). Over the last several decades, significant advances have been made in the treatment of metastatic CRC (mCRC), resulting in improvements in survival (4). The increasing number of effective drugs, together with the improvement of surgical procedures and the availability of different local ablative treatment (LAT), led to a significant increase in overall survival (OS) of mCRC patients, which is now ∼30 months (5).

Anti-epidermal growth factor receptor (EGFR) monoclonal antibodies (MAbs), such as cetuximab and panitumumab, in combination with FOLFIRI or FOLFOX chemotherapies represent a valid option in the treatment of patients with *RAS* wild-type (WT) mCRC (6, 7). While these regimens are currently used in the first- or the second-line setting, in recent years, a few clinical reports have suggested the potential clinical benefit of the re-treatment with anti-EGFR MAbs (8, 9). The reintroduction of the same therapy after a drug holiday have demonstrated activity in a wide range of malignancies. Tumor heterogeneity, the presence of unstable transient resistance mechanisms and ability of cancer cells to mute during treatment could explain this process. Several prospective clinical trials examining anti-EGFR rechallenge are currently underway (9–11).

Immunotherapy has limited efficacy in mCRC. In fact, the use of immune checkpoint inhibitors, such as anti-programmed death 1 (PD-1) or anti-programmed death ligand 1 (PD-L1) MAbs, is clinically effective in the subgroup of patients with microsatellite-instable-high (MSI-H) cancers, but not in the large majority of patients, whose tumors are microsatellite stable (MSS) (12). However, the immune system may play a fundamental role in modulating response to MAb therapies in cancer (13). In this respect, antibody-dependent cell cytotoxicity (ADCC) is enhanced by IgG1 MAbs, such as cetuximab, and may activate both innate and adaptive immune responses (14). Among immunotherapy drugs targeting the PD-1/PD-L1 axis, the anti-PD-L1 IgG1 MAb avelumab has ADCC properties. Preclinical studies have demonstrated that cetuximab may modulate immune response to cancer cells. In fact, cetuximab treatment activates functional cross-talks between natural killer (NK) and dendritic cells, enhances NK cell-mediated ADCC, promotes opsonization of cancer cells by dendritic cells, and increases major histocompatibility complex (MHC) class II molecule expression and recruitment of T cells in the tumor microenvironment. Taken together, these effects may increase cancer cell death, which is induced by cetuximab treatment (13–15).

In this scenario, the inhibition of PD-L1 with the IgG1 MAb avelumab in combination with the anti-EGFR IgG1 MAb cetuximab could be a strategy for potentiating antitumor activity (15). To evaluate this hypothesis, we have conducted a prospective, single-arm, multi-center phase II study of cetuximab plus avelumab as rechallenge treatment in patients with *RAS* WT mCRC in which 77 patients were treated with cetuximab (400 mg/m^2^ and, subsequently, 250 mg/m^2^ weekly) and avelumab (10 mg/kg q14) until disease progression or unacceptable toxicity (16). Seventy-one (92%) patients had MSS cancers, whereas 3 (4%) patients had cancers with high microsatellite instability. Microsatellite status was unknown in 3 (4%) patients. The study met the primary endpoint, with a median OS of 13.1 months [90% confidence interval (CI), 8.0–18.4 months; 44 events]. Median progression-free survival (PFS) was 3.6 months [95% CI, 3.2–4.1 months; 71 events]. Disease control was achieved in 65% patients [95% CI, 53%–75%] with 1 complete response, 5 partial responses, and 44 stable diseases. Grade 3 adverse events were observed in 22% patients, the most common being skin rash (13%) and diarrhea (4%). For 67/77 (87%) patients, baseline analysis of circulating tumor DNA (ctDNA) for *KRAS, NRAS, BRAF*, and EGFR-extracellular domain S492R mutations was feasible. Forty-eight patients had WT disease, whereas a mutation in either *KRAS, NRAS*, or *BRAF* genes was found in the plasma of 19 patients. No EGFR-extracellular domain S492R mutation was found. Patients with *RAS/BRAF* WT cDNA had a median OS of 17.8 months (95% CI, 12.6–23.0 months) compared to 13.8 months (95% CI, 6.6–21.0 months) in patients with mutated ctDNA (hazard ratio, 0.66, 95% CI, 0.33–1.31; *p* = 0.238). Median PFS was 4.1 months (95% CI, 2.9–5.2 months) in *RAS/BRAF* WT patients compared to 3.0 months (95% CI, 2.6–3.5 months) in mutated patients (hazard ratio, 0.42, 95% CI, 0.23–0.76; *p* = 0.003). Disease control was obtained in 35/48 (73%) patients with *RAS/BRAF* WT ctDNA, of which 20 (41%) patients had PFS of 6 months or longer (15). This phase II single-arm clinical trial provided the first evidence of clinical activity of combining cetuximab plus avelumab in 77 patients with *RAS* WT mCRC, who benefited from first-line anti-EGFR-containing therapy and who were re-treated with cetuximab plus avelumab in third or further lines of therapy as rechallenge strategy. Finally, results obtained suggested a new therapeutic option for *RAS* WT mCRC patients (16). Here, we describe the design and rationale for the phase II, randomized CAVE 2 clinical study of the combination of avelumab plus cetuximab as a rechallenge strategy in pre-treated *RAS*, *BRAF* WT mCRC patients treated in first line with chemotherapy in combination with cetuximab and who have had a clinical benefit (complete or partial response) from treatment.

## Methods

### Study Design

This is a non-profit phase II, open-label, randomized clinical study of the combination of avelumab plus cetuximab as a rechallenge strategy in pre-treated *RAS, BRAF* WT mCRC patients treated in first line with chemotherapy in combination with cetuximab and who have had a clinical benefit (complete or partial response) from treatment. The study has been registered at ClinicalTrials.gov (NCT05291156) and has received Italian Drug Agency (AIFA) approval. This trial was also approved by the Ethics Committee of Università degli Studi della Campania Luigi Vanvitelli—Azienda Ospedaliera Universitaria Luigi Vanvitelli—AORN Ospedale dei Colli.

**Figure 1:**
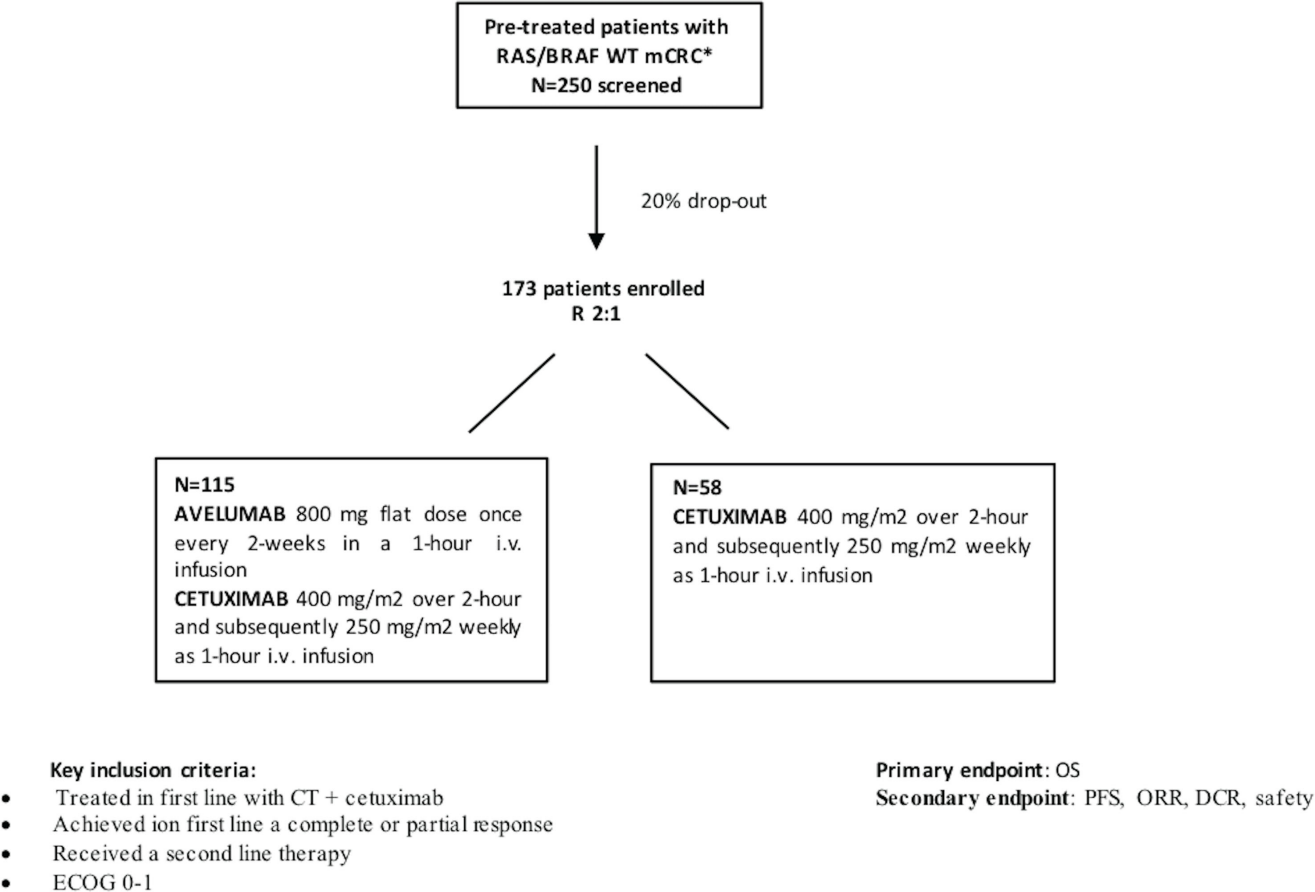
CAVE-2 study design.

The study design is provided in **Figure 1**.

A total of 173 patients will be randomized (2:1) as follows:

• *Cetuximab + avelumab* (115 patients)—cetuximab was administered at 400 mg/m^2^, as a loading dose, and, subsequently, at 250 mg/m^2^ weekly, and avelumab was given intravenously at a flat dose of 800 mg, once every 2 weeks.
• *Cetuximab only* (58 patients)—cetuximab was administered at 400 mg/m^2^ intravenously, as a loading dose, and, subsequently, at 250 mg/m^2^ weekly.

Avelumab will be administered as a 1-h IV infusion at a flat dose of 800 mg every 2-week treatment cycle. Cetuximab will be administered at 1st dose at 400 mg/m^2^ by IV infusion over 120 min. The 2nd dose and subsequent doses will be given at 250 mg/m^2^ by IV infusion over 60 min, every week. The dose of cetuximab will be calculated based on the weight and body surface of the subject determined on the day prior to or the day of each drug administration. Infusion of avelumab will be stopped in case of Grade ≥ 2 infusion-related, allergic, or hypersensitivity reactions (according to NCI-CTCAE v 5). If the subject experiences an infusion-related Grade 2 reaction, the infusion rate of the subsequent administration of avelumab or cetuximab will be reduced by 50%. If the subject has a second infusion-related reaction Grade ≥ 2 on the slower infusion rate, the infusion should be stopped and the subject should be removed from treatment. If a subject experiences a Grade 3 or 4 infusion-related reaction at any time, the subject must discontinue avelumab or cetuximab.

For each patient, before treatment, a blood sample will be obtained and analyzed for circulating free tumor DNA according to NGS (Foundation/Roche), to identify RAS/BRAF WT patients to be enrolled. The same procedure will be performed at progression of the disease (**Table 1**).

**Table 1:** Schedules of assessments.

Measure	Screening/Baseline Assesments	Treatment Phase								Early Discontinuation(x)/Safety Follow-up Visit (X)	Extended Safety Follow-up Visit	Long-term Follow-up
	Day −28 to starting treatment	V1	V2	V3	V4	V5	V6	V7	Until Progression (timeline)	Up to 7/28 Days ( ± 5 days) after Last Treatment	12 Weeks ( ± 2 weeks) after Last Treatment	Every 3 moths ( ± 1 week)
		W1	W2	W3	W4	W5	W6	W7				
		D1	D8	D15	D21	D28	D35	D43				
Written Informed Consent	X											
Collection of tumor tissuewfi 2	X											
Collection of blood sample for RAS, BRAF determination and further analysis (Foundation Liquid CDx)	X									X		
Collection of additional blood/plasma for translational analyses	X									X		
Collection of fecal samples	X									X		
Inclusion/exclusion criteria	X											
Medical history	X											
Demographic data	X											
HBV and HCV testing	X											
Physical examination, including height at screening	X	X	X	X	X	X	X	X	Weekly	x/X	X	
Vital signs	X	X	X	X	X	X	X	X	Weekly	x/X	X	
Weight	X	X	X	X	X	X	X	X	Weekly	x/X	X	
ECOG PS	X	X	X	X	X	X	X	X	Weekly	x/X	X	
Enrollment (if eligible)	X											
Cardiac assessment	X									x/X		
Ophthalmologic assessment	X											
Hematology and hemostaseology	X	X		X		X		X	Every 2 weeks	x/X	X	
Full serum chemistry	X	X		X		X		X	Every 2 weeks	x/X	X	
Urinalysis	X	X		X		X		X	Every 2 weeks	x/X	X	
β-HCG pregnancy test	X	X	X	X	X	X	X	X	Weekly	-/X	X	
Tumor evaluation by CT scan or MRI (a bone scan should be done at screening as clinically indicated)	X							X	Every 8 weeks for 40 weeks and every 12 weeks thereafter	-/X		X
Documentation of AEs and concomitant medications	X	X	X	X	X	X	X	X	Weekly	x/X	X	X
ACTH, ANA, ANCA, RF	X								Week 13, week 25, thereafter if clinically indicated	-/X	X	
T4, and TSH	X								Every 8 weeks	-/X	X	
Pre-treatment and trial drug administration		X	X	X	X	X	X	X	Weekly			

Additional blood/plasma, tumor and fecal samples will be collected and centrally stored for additional translational studies.

Subjects will return to the clinic at regular intervals for assessments. Tumor measurements by computed tomography (CT) scan or magnetic resonance imaging (MRI) will be performed every 8 weeks for 40 weeks and every 12 weeks thereafter to determine response to treatment. Response will be evaluated using the RECIST 1.1.

**Figure 2:**
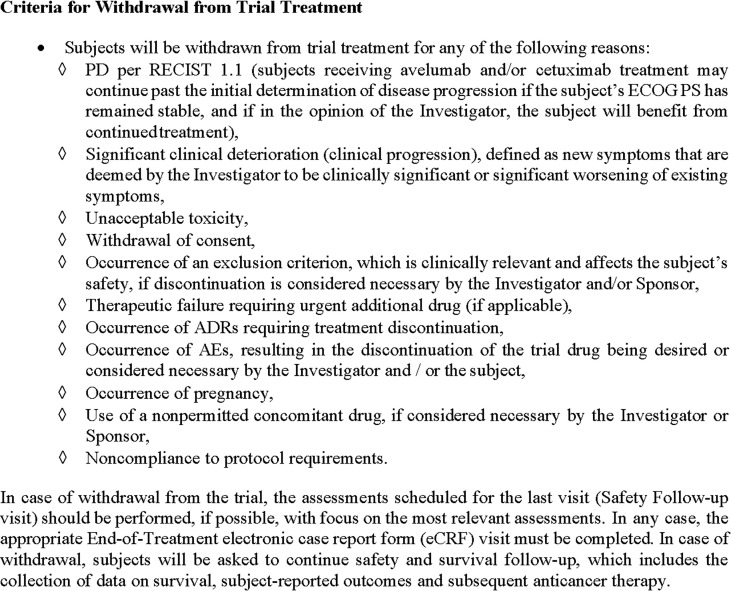
Criteria for withdrawal from trial treatment.

Treatment will continue until:

- disease progression; and
- significant clinical deterioration;
- any criterion for withdrawal from the trial or trial drug is fulfilled (**Figure 2**).

Treatment may continue past the initial determination of disease progression according to RECIST 1.1 if the subject’s performance status has remained stable, and if, in the opinion of the investigator, the subject will benefit from continued treatment and if other criteria are fulfilled as outlined in the protocol, that is, no new symptoms or worsening of existing symptoms and no decrease in performance score.

Subjects will attend clinic visits at regular intervals to receive trial treatment and for efficacy and safety assessments.

**Figure 3:**
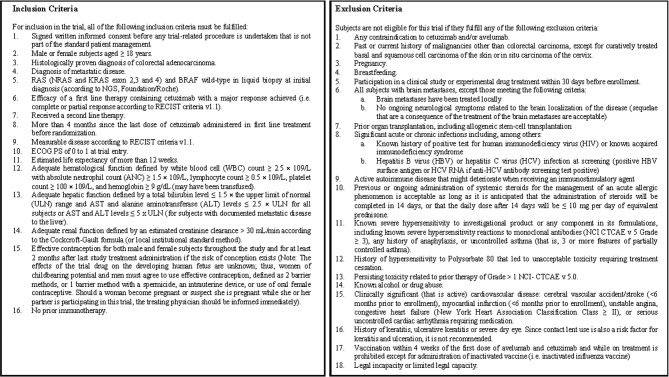
Inclusion and exclusion criteria.

Only persons meeting all inclusion criteria and no exclusion criteria may be enrolled into the trial as subjects. The detailed inclusion and exclusion criteria are provided in **Figure 3**.

### Study Objectives and Endpoints

The primary objective of the study is to evaluate the efficacy (in terms of OS) of avelumab and cetuximab combined in pre-treated *RAS, BRAF* WT mCRC patients compared to cetuximab alone. Secondary objectives are as follows:

• To demonstrate superiority with regard to the objective response rate (ORR) of avelumab and cetuximab combined in pre-treated *RAS, BRAF* WT mCRC patients compared cetuximab alone.
• To demonstrate superiority with regard to PFS of avelumab and cetuximab combined in pre-treated *RAS, BRAF* WT mCRC patients compared to cetuximab alone.
• To determine the safety and tolerability of avelumab and cetuximab combined in pre-treated *RAS/BRAF* WT mCRC patients compared to cetuximab alone.

The primary endpoint for the trial is OS time, defined as the interval from enrollment to death for every cause.

Secondary endpoints will be as follows:

• The ORR according to RECIST 1.1 defined as the proportion of patients who have a partial or complete response to therapy.
• PFS according to RECIST 1.1 defined as the time from random assignment in the clinical trial to disease progression or death from any cause.
• Safety endpoints include AEs, assessed throughout the trial and evaluated using the NCI-CTCAE version 5.0 (CTCAE v 5.0), clinical laboratory assessments, vital signs, and electrocardiogram (ECG) parameters.

Exploratory endpoints include the following: duration of response of cetuximab plus avelumab according to RECIST 1.1 compared to cetuximab alone; EGFR and PD-L1 expression levels in tumor cells as candidate predictive biomarkers; and molecular, cellular, and soluble markers in peripheral blood and/or tumor tissue that may be relevant to the response/resistance to avelumab and cetuximab, including genomic variants, microsatellite instability (MSI) status, tumor mutation burden (TMB), and gene expression signatures.

The analysis on plasma samples will be performed by Foundation Roche laboratories in Germany. The analysis of fecal samples will be performed by the Gastroenterology Unit, Casa Sollievo della Sofferenza, *Via* Padre Pio 7d 70013, San Giovanni Rotondo (FG). Tissue and additional liquid biopsy samples will be tested at the Laboratory of Cell Biology and Biotherapy of the Istituto Tumori di Napoli—IRCCS Fondazione Pascale. The exploratory endpoint analyses will be performed on the ITT analysis set.

Duration of response will be analyzed descriptively by treatment arm. The Kaplan–Meier estimate of median time along with its 95% CI will be calculated for the duration of response.

### Trial Procedures and Assessments

#### Screening/Baseline Assessments

During the screening period and before any trial-related investigations and assessments are started, subjects will be asked to sign the informed consent form (ICF). The screening procedures and baseline assessments will be completed within 28 days from signing the ICF (i.e., 28 days before start of treatment). Failure to establish eligibility within 28 days would result in screening failure and the subject will be excluded from the trial; however, subjects can be re-entered in the trial based on the investigator’s judgment within 6 weeks of signing the ICF. In this case, a new ICF will be required to be signed by the subject.

For the trial entry, all of the subjects must fulfill all inclusion criteria described in **Figure 3**, and none of the subjects should have any exclusion criteria from the list described in **Figure 3**.

The assessments and procedures described in this section must be performed during the screening period.

In order to determine the subject’s eligibility to the trial, a complete medical history of each subject will be collected and documented during screening. The tumor disease information regarding mCRC history will be documented and verified at the screening visit for each subject. Vital signs including body temperature, respiratory rate, heart rate (after a 5-min rest), and arterial blood pressure (after a 5-min rest) will be recorded at trial entry.

A physical examination (including, in general, appearance, dermatological, head/neck, pulmonary, cardiovascular, gastrointestinal, genitourinary, lymphatic, musculoskeletal system, extremities, eyes [inspection and vision control], nose, throat, and neurologic status) will be performed and the results will be documented. The ECOG PS will be documented during the screening phase and at each scheduled visit. Body weight and height (screening only) will be recorded. Blood samples will be collected at screening for clinical laboratory parameter evaluations. These clinical laboratory test results will not only serve as the baseline values for subsequent safety clinical laboratory evaluations during the trial, but also help to make sure that each enrolled subject fulfills all the trial entry criteria and does not meet any of the trial exclusion criteria for laboratory parameters.

The screening laboratory examination includes hematology, hemostaseology, full serum chemistry (including core chemistry), and full urinalysis (dipstick plus microscopic evaluation). Adrenocorticotropic hormone (ACTH), ANA, ANCA, rheumatoid factor (RF), free thyroxine (T4), and thyroid-stimulating hormone (TSH) will also be assessed at screening for all subjects. Additionally, HBV surface antigen and anti-HCV tests must be performed at screening to exclude hepatitis infection. If the anti-HCV antibody test is positive, infection should be confirmed by an HCV RNA test.

A serum β-human chorionic gonadotropin (β-HCG) pregnancy test will be performed for female patients of childbearing potential. Female patients who are postmenopausal (age-related amenorrhea ≥ 12 consecutive months and increased follicle-stimulating hormone [FSH] >40 mIU/ml) or who have undergone hysterectomy or bilateral oophorectomy are exempt from pregnancy testing. If necessary to confirm postmenopausal status, FSH will be drawn at screening.

A 12-lead ECG will be recorded at screening and at the early discontinuation/safety visit after the subject has been in a supine position breathing quietly for 5 min.

Baseline imaging will be performed within 28 days prior to the start of treatment in order to establish baseline disease status of target and nontarget lesions according to RECIST 1.1. Acceptable modalities include CT scans (chest, abdomen, and pelvis), CT chest with contrast together with MRI of the abdomen and pelvis, or positron emission tomography/CT scans. A brain CT/MRI scan is required at screening if one has not been performed within 8 weeks prior to starting treatment. In general, lesions detected at screening/baseline need to be followed using the same imaging methodology and preferably the same imaging equipment at subsequent tumor evaluation visits.

For each patient, before treatment, a blood sample will be obtained and analyzed for circulating free tumor DNA according to NGS (Foundation/Roche); this assay will allow us to define *RAS/BRAF* WT patients to be enrolled.

FFPE tumor blocks (or 10 slides of conventional thickness and polarity for IHC and 10 slides of 10-micron thickness for molecular biology) of primary and/or metastatic sites should be collected for each enrolled patient. In addition, approximately 1 g of fecal samples will be collected before treatment.

#### Treatment Period

Treatment phase begins the day of first infusion and ends when a decision is made to stop the trial drugs by the investigator or when consent is withdrawn by the subject.

Visits will take place every week ( ± 1 day).

The main assessments are as follows:

- Tumor responses will be assessed every 8 weeks ( ± 5 days) from starting treatment for 40 weeks and every 12 weeks ( ± 5 days) thereafter, per RECIST 1.1 while on trial.
- Vital signs will be collected prior to each trial drug administration. Administration of trial drugs will take place only after relevant results have been checked by a medically qualified person.
- Blood chemistry and hematology assessments must be performed at baseline, every 2 weeks ( ± 2 days) prior to each avelumab plus cetuximab or cetuximab dose, at the early termination visit, and at 28 days ( ± 5 days) post-treatment safety follow-up visit.
- Urine pregnancy test for women of childbearing potential must be performed at baseline and at least every month during treatment.
- Free T4 and TSH must be performed at baseline and at least every 8 weeks ( ± 5 days) during treatment and at the early discontinuation visit or 28 days post-treatment safety follow-up visit (if not performed in the previous 8 weeks).
- AEs and concomitant medications will be documented at each visit.

In the combination arm, avelumab treatment will be administered by IV infusion once every 2 weeks whereas cetuximab treatment will be administrated by IV infusion once every week until disease progression, significant clinical deterioration (clinical progression), discontinuation for unacceptable toxicity, or withdrawal of consent. In the cetuximab arm, cetuximab treatment will be administrated by IV infusion once every week until disease progression, significant clinical deterioration (clinical progression), discontinuation for unacceptable toxicity, or withdrawal of consent.

Note: Treatment may continue past the initial determination of disease progression per RECIST 1.1 if the subject’s ECOG PS has remained stable, and if, in the opinion of the investigator, the subject will benefit from continued treatment and if other criteria are fulfilled as outlined in the protocol, that is, no new symptoms or worsening of existing symptoms and no decrease in performance score.

#### Early Discontinuation Visit

Any subject who experiences an AE that mandates discontinuation of trial treatment should have a discontinuation visit within 7 days of the decision to discontinue trial treatment.

The main assessments are as follows:

- A physical examination including vital signs, body weight, 12-lead electrocardiogram (ECG), and a determination of the Eastern Cooperative Oncology Group Performance Status (ECOG PS).
- Safety laboratory assessments.
- AEs and concomitant medications assessments.

Once the early discontinuation visit has been performed, subjects must return for the safety follow-up visit within 28 days (± 5 days) after the last administration of trial treatment or before the start of any antineoplastic therapy, whichever occurs earlier.

#### Follow-Up Phase

The follow-up phase starts when the decision to stop the trial drug treatment has been made.

Subjects will have:

- a safety follow-up visit at 4 weeks (± 5 days) after the last administration of trial treatment or before the start of any other antineoplastic therapy.

- an extended safety follow-up visit 12 weeks (± 2 weeks) after the last administration of trial treatment. Given the potential risk for delayed immune-related toxicities, safety follow-up must be performed up to 90 days after the last dose of avelumab administration.

The extended safety follow-up beyond the 12-week safety follow-up visit may be performed either *via* a site visit or *via* a telephone call with subsequent site visit requested in case any concerns are noted during the telephone call.

- a survival follow-up period (every 3 months ± 1 week), after the extended safety follow-up visit, for survival assessment (including assessment of any further tumor therapy).

After the safety follow-up visit, only treatment-related AEs have to be documented until the extended safety follow-up visit.

Subjects with a serious AE (SAE) ongoing at the extended safety follow-up visit must be followed up by the investigator until stabilization or until the outcome is known, unless the subject is documented as “lost to follow-up.”

Subjects who discontinue the trial treatment for reasons other than disease progression according to RECIST 1.1 will be followed up every 8 weeks ( ± 5 days) for radiographic assessment from starting treatment for 40 weeks and every 12 weeks ( ± 5 days) thereafter, per RECIST 1.1, until disease progression, lost to follow-up, or withdrawal of informed consent.

The survival follow-up will continue until 12 months after the last subject has been randomized, followed by an additional long-term follow-up period of 12 months.

### Statistical Analysis

The primary endpoint of the trial is the OS, defined as the time (in months) from date of starting treatment to the date of death, regardless of the actual cause of the subject’s death.

The study aims to demonstrate a median OS of 10 months for cetuximab and 15 months for cetuximab plus avelumab, which corresponds to an improvement of 33% in median OS (hazard ratio 0.67). To obtain a power of 90%, with one-sided alpha 0.20 and a randomization ratio of 2:1, 123 events are needed for the analysis. If 173 patients (115 cetuximab + avelumab and 58 cetuximab alone) will be accrued in 24 months, the number of events needed for analysis should be obtained after an additional 12 months. We have considered approximately 250 patients to screen with basal liquid biopsy test (Foundation One Liquid CDx) to finally enroll 173 *KRAS, NRAS, BRAF* WT patients. A potential dropout of 20% has also been considered. The Kaplan–Meier method will be used to estimate PFS and OS. Statistical analyses will be performed using the SPSS package (v.23). Planned accrual period: 24 months, further follow-up: 12 months, followed by an additional long-term follow-up period of 12 months. For subjects who are still alive at the time of data analysis or who are lost to follow-up, OS time will be censored at the last recorded date that the subject is known to be alive (date of last contact, last visit date, date of last trial treatment administration, or date of last scan, whichever is the latest) as of the data cutoff date for the analysis. If the date of last known status of alive or death date is after the data cutoff date, subjects will be censored at the data cutoff date.

Secondary efficacy analyses will be performed on the ITT analysis set. For the secondary endpoint analysis of PFS time according to RECIST 1.1, the statistical analysis will be the same as described for the primary analysis of OS time. For the secondary endpoint analysis of ORR according to RECIST 1.1, the ORR in terms of having a confirmed BOR of CR or PR will be calculated along with the corresponding two-sided exact Clopper-Pearson 95% CIs.

### Dissemination Plans

The results will be published in a paper after the completion of the study.

### Trial Status

Protocol version: v2.0 was finished on February 2022. Participant recruitment will start in May 2022 and is expected to end in May 2025.

## Discussion

The introduction of target therapy and the development of continuum of care strategies profoundly changed the treatment landscape for patients with unresectable mCRC, deviating from accounting only for the sidedness of the primary tumor, performance status, volume of disease, and potential resectability to also include the genetics of the tumor and to support the use of precision oncology for treatment decision (3–5). Although new therapies to further extend survival and quality of life in patients with mCRC are needed, better selection of patients for available treatment choices is also essential.

Recently, immunotherapy using an anti-checkpoint antibody, anti-PD-1, has achieved impressive results in patients with MSI tumors (12). Therefore, MSI is an attractive biomarker for immunotherapy. Although MSI cancers are known to be associated with increased tumor-infiltrating lymphocyte (TIL) density, the nature of the immune infiltration and the molecular drivers of the immune phenotype in MSS CRC are poorly understood.

Several efforts have been made in order to improve immunotherapy response in the MSS subtype. The combination of immunotherapies with different target agents or conventional chemotherapeutic strategies might represent a useful and practical means to stimulate immune cell infiltration and elicit immune response (13). To this purpose, several clinical trials are investigating the combination of immune-based agent with a target agent.

Preclinical data suggest that cetuximab is able to induce an innate immune effector function *via* the activation of NK cells (14). The Fc constant region of cetuximab binds to the activating receptor CD16/FcγRIII on NK cells, leading to NK cell activation and subsequent lytic activity on tumor cells by a process called ADCC (14). NK cells play a key role in cancer immunotherapy, possessing specific antitumor activities in the presence of tumor antigen-targeting MAbs through ADCC. Avelumab is a fully human IgG1 with a dual mechanism of action. It blocks the interaction between PD1 and PD-L1 and is able to enhance NK cell-mediated ADCC (15).

Thus, the combination of avelumab and cetuximab may result in synergistic activity, increasing the efficacy of anti-EGFR and anti-PD-L1 activity (15).

Several clinical trials investigating the combination of cetuximab and avelumab are already in progress in several cancer types such as patients with squamous cell carcinoma of the head and neck (SCCHN) and mCRC.

In the safety phase of the randomized phase III trial GORTEC 2017-01 (REACH), treatment with avelumab in combination with RT-cetuximab has been compared with standard of care (SOC) cisplatin-RT and/or to SOC RT-cetuximab alone as first-line treatment in locally advanced (LA) squamous cell carcinoma of the head and neck patients (17). This trial includes 2 different cohorts of patients: deemed fit (cohort 1) to receive high-dose cisplatin (CDDP 100 mg/m², every 3 weeks) or unfit to receive a standard dose of CDDP (cohort 2). The SOC treatment is intensity-modulated radiotherapy (IMRT, 69.96 Gy, 33 fractions) combined with CDDP in cohort 1 and with cetuximab in cohort 2 (400 mg/m² day 7 and 250 mg/m² weekly). In both cohorts, experimental arms are IMRT concomitant with cetuximab (same schedule as in SOC) and avelumab (10 mg/kg day-7 and every 2 weeks) followed by 10 mg of avelumab. Between 2017 and 2020, 707 patients were randomized. After a median follow-up of 21 months, a favorable effect of adding avelumab to cetuximab was seen on the primary endpoint, PFS, local-regional control, and distant metastases (16).

In mCRC, AVETUX, a single-arm multicentric phase II trial, evaluated the combination of cetuximab and avelumab with chemotherapy (FOLFOX) as first-line treatment *in RAS/BRAF* WT MSS or MSI mCRC patients (18). This combination regimen showed a high response rate of 79.5% with 6 CR and 25 PR. Disease control rate was 92.3% and mPFS was 11.1 months (18). The treatment was well tolerated with no addition of any unexpected AEs to the safety profile of the standard cetuximab + FOLFOX regimen.

The combination of avelumab + cetuximab + irinotecan is being examined in the AVETUXIRI trial in patients with *BRAF* WT MSS mCRC who are refractory to standard chemotherapy and anti-EGFR treatment (19). The trial includes two cohorts: cohort A with *RAS* WT mCRC and cohort B with *RAS* mutated patients. The 2 primary endpoints were overall response rate, defined as either a CR or a PR according to immune RECIST (iRECIST), and safety. As of January 2020, 23 patients had been enrolled and received treatment. Three patients in cohort A achieved a PR, and none achieved a CR or PR in cohort B. No unexpected safety events occurred. Median PFS was 4.2 months in cohort A and 3.8 months in cohort B; median OS was 12.7 months and 14.0 months, respectively (19).

Moreover, FIRE-6 trial (EudraCT 2018-002010-12) is investigating the combination of avelumab and cetuximab with the FOLFIRI regimen, followed by maintenance with avelumab, in *RAS* and *BRAF* WT untreated mCRC. Finally, the AVETRIC trial (EudraCT 2019-0041501-24) is investigating the combination of avelumab + cetuximab + FOLFOXIRI in patients with *RAS* WT mCRC.

We have conducted a prospective, single-arm, multi-center phase II study of cetuximab plus avelumab as a rechallenge treatment in patients with *RAS* WT mCRC in which 77 patients were treated with cetuximab (400 mg/m^2^ and, subsequently, 250 mg/m^2^ weekly) and avelumab (10 mg/kg q14) until disease progression or unacceptable toxicity (16).

Based on the hypothesis of a synergistic effect and on the safety data available from this trial, cetuximab in combination with avelumab could be a valid therapeutic option as a third-line rechallenge treatment for mCRC *RAS* WT patients that achieved a major response to cetuximab first-line therapy.

Rechallenge with EGFR inhibitor in patients who responded to first-line treatment containing an EGFR-based agent is emerging as a valid therapeutic approach in mCRC patients (8).

The analysis of ctDNA will reveal patients who had no detectable *RAS* mutations at the time of rechallenge, and it highlights the actual reliability of liquid biopsy as a tool to inform therapeutic decisions. In particular, the use of a large NGS panel such as FoundationOne could help to identify potential resistant mechanisms to anti-EGFR-based treatments in order to better select patients that could benefit from a rechallenge treatment (20).

From this intuition and based on promising results obtained in the CAVE trial, the CAVE-2 clinical trial emerged as a novel therapeutic option. We expect that the results of this phase II study will provide preliminary evidence for further evaluation of this combination treatment option in patients with *RAS* WT mCRC to be included in clinical practice.

Our findings also bear some relevance to the evolving field of immunotherapy, and in the attempt to improve immunotherapy response in contexts different from the MSI-H subset.

Finally, this research will substantially contribute to our understanding of the genetic and molecular bases of resistance to target therapies in CRC and to validate a new approach to overcome drug resistance.

## Data Availability Statement

The original contributions presented in the study are included in the article/supplementary material. Further inquiries can be directed to the corresponding author.

## Ethics Statement

The studies involving human participants were reviewed and approved by the Ethics Committee of Università degli Studi della Campania Luigi Vanvitelli—Azienda Ospedaliera Universitaria Luigi Vanvitelli—AORN Ospedale dei Colli. The patients/participants provided their written informed consent to participate in this study.

## Author Contributions

FC, EvM, and SN developed the study concept and protocol. SN, GM, DC, EvM, and TT have written the manuscript for the study protocol with the support of MM, NN and AA. All authors contributed to the article and approved the submitted version.

## Funding

This was an academic nonprofit study, conducted in the GOIM clinical research network. Two research grants that partially covered the costs of study were provided by Merck and Regione Campania (I-Cure Research Project, Grant number: Cup 21C17000030007).

## Conflict of Interest

The authors declare that the research was conducted in the absence of any commercial or financial relationships that could be construed as a potential conflict of interest.

## Publisher’s Note

All claims expressed in this article are solely those of the authors and do not necessarily represent those of their affiliated organizations, or those of the publisher, the editors and the reviewers. Any product that may be evaluated in this article, or claim that may be made by its manufacturer, is not guaranteed or endorsed by the publisher.
